# It is Not Too Late for Reconciliation Between Israel and Palestine, Even in the Darkest Hour

**DOI:** 10.1007/s11673-024-10347-x

**Published:** 2024-03-22

**Authors:** P. A. Komesaroff

**Affiliations:** Monash University, Melbourne, Australia

**Keywords:** War, Reconciliation, Gaza, Israel, Holocaust, Peace, Conflict, Dialogue, Ethics

## Abstract

The conflict in Gaza and Israel that ignited on October 7, 2023 signals a catastrophic breakdown in the possibility of ethical dialogue in the region. The actions on both sides have revealed a dissolution of ethical restraints, with unimaginably cruel attacks on civilians, murder of children, destruction of health facilities, and denial of basic needs such as water, food, and shelter. There is a need both to understand the nature of the ethical singularity represented by this conflict and what, if any, options are available to allow the reconstruction of communication between the warring parties. This article seeks to address these tasks by analysing the conflict as inherently an ethical one, in the sense that it exposes a rupture in the fabric of communicative relationships that has evolved systematically out of the deep cultural structures from which all protagonists have emerged. Drawing on the work of Levinas, Habermas, Arendt, and others, and referring to the specific circumstances in the region, it examines the ethical sources of the crisis and tries to identify conditions for its resolution. The possibility of reconciliation—that is, of refiguring relationships to open up a space for dialogue to create pathways to heal the ruptures—is examined. The dark legacy of the Holocaust is identified as an abiding cultural vulnerability for both societies. It is concluded, however, that the rich history of partnerships and collaborations between Jews and Palestinians provides a robust infrastructure on the basis of which a sustainable peace might be built, providing a much-needed source of hope.

The conflict in Gaza and Israel that ignited on October 7, 2023 signals a catastrophic breakdown in the possibility of constructive dialogue between Palestinians and Israeli Jews. In addition to producing heavy civilian casualties, it has exacerbated the deep pre-existing divisions, further eroding what little existed of trust, and inflaming bitter feelings of mutual hatred and betrayal. Indeed, the breakdown is much worse than this. The actions on both sides have revealed a dissolution of ethical restraints, with unimaginably cruel attacks on civilians, murder of children, destruction of health facilities, and denial of basic needs such as water, food, and shelter.

Alongside the strident calls for vengeance an impression has formed that following the shattering of what had appeared as a stable, if by no means peaceful, equilibrium between the Israeli army and the Palestinian resistance any hope for a definitive, sustainable process of reconciliation between the warring parties has now also gone. While for many, the prospects for a resolution to the conflict does indeed seem more hopeless than ever, in this essay I argue that, even in these dark days, there nonetheless remain reasons for qualified optimism about a possible future pathway to reconciliation. Drawing on historical experiences and the vast array of partnerships, associations, collaborations, and friendships that have been built up over the years, I seek to identify traces of a route whereby a space for a productive dialogue that enables fundamental structural changes may one day be reconstructed. I write as someone with a deep affection for both the Jewish and Palestinian peoples who has spent many years trying to support enhanced communication and the formation of trusting relationships across the region. In addition to personal experiences, I draw on extensive published materials, my own and others’ observations, and interviews with active participants on all sides.^1^

Notwithstanding the bare threads of optimism it is, of course, hardly possible to overstate the magnitude of the challenge to secure peace across the region. Further, the transformation of agreements that had seemed to be within grasp into hopeless mirages is an all too familiar experience, and current political discourses are naturally preoccupied with ending, or even merely pausing, the killing. Nonetheless, the unthinkable prospect of another catastrophe is sufficient to infuse the sadly depleted peace process with a melancholy and resolute urgency.

## The First Casualty of War is Ethics

The key thesis of this article is that the problem underlying the conflict in Israel and Palestine is inherently an ethical one—not in the sense that there is one-sided moral culpability but rather that there has been a rupture in the fabric of communicative relationships that has evolved systematically out of the deep cultural structures from which all protagonists have emerged. This rupture is presently so profound that it has led to the dissolution of the most basic constraints on actions between individuals and communities, leading to levels of violence and cruelty previously unimaginable.

The task of reconciliation—if it is possible at all—is to respond to this ethical collapse by seeking to refigure relationships, with the aim of opening up a space for an exploratory, creative process of dialogue within which pathways may become possible to heal the ruptures that have so tragically opened up. This is not a new task: indeed, it has many antecedents, some of which have delivered modest, if transient, success (Webster 2018; Celermajer 2009; Avruch and Vejerano 2001). Nonetheless, major challenges remain, not least those of diagnosing and then reversing the forces that generated the present ethical calamity.

In the usage adopted in this article, explained below, “ethics” is not limited to narrow formulations of normative rules or theories but refers more broadly to the bond that underlies, and is the condition of possibility of, all sociality—the primordial connection between people across cultures, ethnicities, genders, nationalities, philosophical dispositions, and religions. As I will discuss, this perspective on ethics draws on the philosophy of the French Jewish philosopher Emmanuel Levinas and other contemporary thinkers. The ethical bond referred to is the generative source of language and the dialogue that emerges from it, and the condition of possibility for communicative interactions that generate shared meanings, theories, and the concept of truth itself.

It is often said—supposedly quoting Aeschylus—that “the first casualty of war is truth.” This claim, however, is not strictly correct: in reality, war’s first casualty is not truth but ethics, or more precisely, the ethical bonds which form the bedrock on which truth is founded. Indeed, as a matter of definition, wars occur because non-violent processes for resolving conflict have collapsed, leaving the explosion of violence and the brutality that goes with it the only remaining “communicative” resource. This cutting off of dialogue is linked immanently to the fracturing of the ethical bond and thereby to the dissolution of shared meanings and of discourse directed at knowledge and truth. This has palpably occurred repeatedly in Israel and Palestine, where failed promises of cooperation and friendship have so often exploded into mutual terror, gratuitous murder, and other atrocities.

For the same reasons the equally famous adage—by Carl von Clausewitz—that “war is the continuation of politics by other means” is also mistaken. Indeed, here the exact opposite is the case. War entails the *dissolution* of civil politics and the communicative discourses that are central to it. The space that remains becomes a place of violence and cruelty where value-based rules of conduct cease to exercise a regulatory force. Despite the affected righteousness of the familiar talk about “rules of war,” these are almost invariably disregarded by combatants whenever it is convenient for them to do so. Similarly, accusations of “war crimes” and “crimes against humanity,” and calls for the exercise of a “responsibility to protect,” are at best selectively deployed, or worse, are used as weapons in the conflict itself. Accordingly, the various mechanisms developed to regulate violent conflicts in many cases do little to impose order on the chaos of war. For a conflict to end, or to be avoided, the critically disrupted communication has to be restored so that dialogue can be resumed and non-violent options devised to replace them, however painstaking that task may be. It is this therapeutic process of restoration that is referred to as “reconciliation.”

## There is a Critical Need for Reconciliation in Israel and Palestine

It is hard to contest that an active process of reconciliation is urgently needed in Israel and Palestine—that is, the development of engagements between the parties in conflict that support the re-establishment of respectful communication, constructive relationships, and the reforming of the ethical bond that has been shattered by the conflict.

Reconciliation in this sense involves the pursuit of dialogue in which differences in cultural, ethical, religious, and other values can be negotiated. The purpose cannot be the elimination of such differences but rather their acknowledgment and the ability to use them as a basis for mutual respect. In order to make sense of differences in this way a common starting point is still necessary. As pointed out by the philosopher Michel Serres, “communication is only possible between two persons used to the same forms, trained to code and decode a meaning by using the same key” (1968, 65). That is, there must be a common antecedent infrastructure on the basis of which the communication can proceed. However, once underway, the trajectory marked out by the discourse may move in unexpected directions, either bringing the protagonists closer together or taking them further apart.

In the Israel-Palestine case, despite the current ethical rupture, it is not difficult to identify a common bedrock of values and aspirations on which such a dialogue can build. These have been described many times. At the most basic level they encompass the shared experiences of thousands of years of history and the aspirations arising in the overlapping life-worlds of all citizens in the regions—including the hopes of people everywhere for access to the material means of existence, along with the conditions for safe and fulfilling lives for their children, free from the threats of physical violence and psychological trauma, and the ability to pursue sensual pleasures, and intellectual, aesthetic, or religious practices as desired (Dowty 2012; Lerner 2011; Caplan 2019).

However, the existence of shared hopes and aspirations is itself obviously not sufficient to guarantee the possibility of mutual understanding, especially because, along with the common values there are also profound differences in worldviews and social and cultural practices—linked, of course, to underlying economic, political, and cultural structures. Indeed, often the disjunction is so profound that the worlds of meaning and sense in which the opposing parties operate are so different that it may seem that there is no possibility of meaningful communication between them at all—that is, in formal terms, where the discourses appear to be incommensurable (Feyerabend 2020; Komesaroff 2014). The hypothesis underlying the theory and practice of reconciliation is that this is never the case. Rather, some communicative engagement, a mutual making sense, is always possible. The construction of the machinery to allow this to occur is precisely the job of reconciliation.

It is important to emphasize that reconciliation conceived in this way is conceptually distinct from “conflict resolution,” the aim of which is to overcome key differences between parties in order to overcome or annul conflicts. Reconciliation actually proceeds in the opposite direction. As an active process of translation across disparate systems of meaning, it does not involve, or aspire to, the overcoming or dissolution of the multiple complex differences that separate the antagonists. On the contrary, it accepts these differences, not as obstacles to peace but as rich resources from which novel possibilities and opportunities can be forged. Drawing on them, it seeks to fashion a restoration of the capacity for dialogue, through which the substantive aspects of the conflict can be projected into a shared space of meaning where, through a process of analysis, reflection, negotiation, and compromise, processes and practices can be fashioned that permit the parties to live and work together in a dynamic equilibrium.

In conventional discourses about the Israel-Palestine conflict, the most frequently proposed route to a solution is the so-called “two-state” model, in which two sovereign entities come into existence in geographical proximity, providing support and protection to both parties and a process for regulating relations between them. While such an outcome would undoubtedly be highly desirable, and arguably some form of a two-state solution is ultimately the only possible way forward, recent experience has exposed the many obstacles it faces, including the historical enmeshment of the populations and the need for active cooperation in the legal, cultural, religious, environmental, and educational domains. More fundamentally, it is evident that, to be sustainable, such an outcome would need to represent more than a purely formal political arrangement imposed from above but would require an individual- and community-based process for healing the deep historical wounds that have been allowed to fester for so many years. Arguably, the failure of the Oslo Accords, which were signed in 1993 and ultimately collapsed in 2002 following vigorous and implacable opposition from within both Palestinian and Jewish populations, provides proof of this (Falah 2021).

Reconciliation is not an alternative to a political or diplomatic solution but a condition of its possibility. No attempt at resolving the conflict solution could possibly be viable in the absence of an infrastructure for peace with deep roots in both communities. The piecing together of such an infrastructure is precisely the work of reconciliation. It includes not “peace plans,” “blueprints,” “road maps,” or other grand schemes grandiosely conceived by parties external to the experience of the conflict and the boundless suffering it has engendered but a painstaking reconstruction of trust that can form the foundation on which mutually respectful dialogues can be founded. Such trust can only come into existence through the enactment of actual interactions and exchanges that bring individuals together around common values that are jointly encountered in shared lifeworld experiences. If allowed to proceed, such a healing process will facilitate shifts away from polarized positions that annul or demonize the members of the opposing group and engender acknowledgment of cultural, religious, and other forms of difference in an unfolding, dynamic process of dialogue.

Of course, the restoration of dialogue is never straightforward and itself requires both conditions and specific contents, as will be described below. In the current setting, for many, this may seem completely beyond grasp. The depth of distrust, hostility, and antipathy—indeed, of overt hatred—is greater than many people can recall. The opposing positions are so polarized it is hard to discern common ground on which to start a reconstruction process. Rather than harmony and cooperation, bitter vengeance and strident calls for inflicting pain on the enemy are the focus of polemics of leaders on both sides. Indeed, we are presently so far from respectful dialogue that there are words or sentences that are difficult to articulate within communities on either side. For example, in Israel it is difficult to opine publicly that the fury of October 7 needs to be understood in the context of the years of cruelty and oppression of Palestinians, while in Palestine proposals for alliances or dialogues with sympathetic Israelis may be sufficient to attract threats and accusations of outright treason.^2^

## All Ethics is Founded on Communication

As forbidding as the obstacles are, some mechanism must be found to allow a passage out of the impasse. Ultimately blockages in communication must be overcome if social conflicts are to be resolved or, at least, if peaceful processes are ever to replace the violent ones. To achieve this, it is necessary to understand the structure and dynamics of communication and the specific conditions that may corrupt or undermine it in a particular conflict setting. The theory of communication is by no means straightforward but, together with its entwining with the problems of ethics, has been the subject of a great deal of work by many thinkers.

This fundamental and originary role of dialogue has in fact been the subject of investigation by many modern thinkers.^3^ An early powerful formulation, which aptly illustrates what is at stake, was provided by the Russian linguist and literary theorist Mikhail Bakhtin, who proclaimed that dialogue encapsulates “the nature of human life itself.” In dialogue, he claimed, “a person participates wholly and throughout his whole life: with his eyes, lips, hands, soul, spirit, with his whole body” (Bakhtin 1984, 293). Referring to the contemporary language of linguistic theory, the philosopher Jurgen Habermas explicitly draws the link with ethics:

Speech acts … serve to produce (or renew) interpersonal relationships … And they serve to express lived experience, … Thus agreement in the communicative practice of everyday life rests simultaneously on intersubjectively shared propositional knowledge, on normative accord and on mutual trust. (Habermas 1990, 236)

In order to elaborate in detail the links between the practical problem of disrupted communication and the theoretical one of an ethical rupture, Habermas analysed both the dynamics of communication and the social interactions associated with it—what he referred to as “communicative action”—and the pathologies that disrupt its functioning (Habermas 1985). This work was developed in relation to a large and complex body of theory about the nature of communication, language, and ethics in the formation and regulation of the institutional formations of economy and culture. Arguing in support of a fundamental role for the standards of rational discourse to regulate communicative interactions, he claims:

The strength of a consensus brought about in unconstrained communication is not measured against any success but against the claim to rational validity that is immanent in speech … We allow ourselves to be convinced of the truth of a statement, the rightness of a norm, the veracity of an utterance; the authenticity of our conviction stands and falls with belief, that is, with the consciousness that the recognition of those validity claims is rationally motivated. (Habermas 2017)

Using the technical language of contemporary linguistics developed by Austin (1962), Habermas argues that language itself contains forces which, if relieved of contaminating distortions, inherently and immanently allows a rationally motivated consensus to unfold:

In short, the communicatively produced power of common convictions originates in the fact that those involved are oriented to reaching agreement and not primarily to their respective individual successes. It is based on the fact that they do not use language “perlocutionarily,” merely to instigate other subjects to a desired behaviour, but “illocutionarily,” that is, for the noncoercive establishment of intersubjective relations. (Habermas 2017)

In an analogous and strikingly consistent manner, the Jewish political theorist, Hannah Arendt, investigated the conditions that gave rise to the crimes of Nazism, the most egregious, catastrophic breakdown in the ethical regulation of social relationships of the twentieth century. She too analysed the form of intersubjectivity generated in the praxis of speech as a basic feature of cultural life. Thereby, in a manner somewhat analogous to that of Habermas, she displayed how intersubjectively shared meanings across life-worlds are formed in the medium of communicative action.

For Arendt, the “human condition” was characterized by the three paradigmatic activities of “labour”, “work,” and “action.” It is the last of these—action—that defines the unique nature of human existence because it is the domain within which meanings are produced that are not required for pure existence. The medium of action includes speech, interaction, and engagement with others, in a context of “plurality” or distinctness and specificity of individual agents. As for Habermas, the communicative exchange that arises here is the domain of debate around meanings, values, truth, knowledge, political goals. It is the ground of ferment, of creative difference, and freedom. For Arendt, speech and action reveal the unique distinctness of human beings:

Through them, men distinguish themselves instead of being merely distinct, they are the modes in which human beings appear to each other, not indeed as physical objects, but qua men. (Arendt 2013, 176)

With word and deed, we insert ourselves into the human world, and this insertion is like a second birth, in which we confirm and take upon ourselves the naked fact of our original physical appearance. (Arendt 2013, 176)

In her work *The Human Condition* Arendt stresses repeatedly that, understood in this way, action is inherently symbolic in character and that the web of human relationships is sustained by communicative interaction (Arendt 2013, 178–179, 184–160). As for Habermas, she sees the domain of communicative action simultaneously as the site of novel meaning construction, ideas, values, shared goals enacted via actual life-world partnerships and, at times, subject to disabling pathologies, which can not only obstruct but also undermine and even threaten its very conditions of possibilities. While Habermas developed a theory of the systems discontinuities to which the public sphere was subject, in her work *The Origins of Totalitarianism*, Arendt mapped out a nosology of the pathologies that generated totalitarianism and National Socialism.

For both, the political objective was to elaborate the conditions that would allow such pathologies to be identified and circumvented, in order to support conditions where, as Habermas puts it, “word and deed have not parted company, where words are not empty and deeds not brutal, where words are not used to violate and destroy but to establish relations and create new realities,” where the catastrophic collapse of communication that “cuts off the public exchange of opinions, degenerates to a rule based on violence” can be avoided (Habermas 1977, 9–10; see also Habermas 1985).

Communication in social life is always vulnerable to distortions and blockages, and the threat of a collapse into violence is always present. It is the task of social actors seeking to avoid violent conflicts and maintain stability of social institutions to identify the sources of impaired communication and to construct therapeutic techniques to circumvent them. The latter may take many forms: they may involve rigorously controlled educational programmes, facilitated conversations via social or mass media, or informal life-world engagements around shared interests in culture, sport, environmental protection, health and well-being etc. In a particular setting, exactly how to implement a healing discourse may be uncertain and controversial. Habermas’ own commitment to the overwhelming importance of the unconstrained flourishing of rational dialogue has been regarded by many as inadequate in the face of the complexities of modern societies and the multifaceted nature of communication and cultural expression. Nonetheless, his admonition to explore other discursive settings—such as medicine, psychology, and educational theory—in search of resources for the development of such therapies has proved highly fecund.

## Ethics, Reconciliation and Communicative Action are Intertwined

Habermas and Arendt and many other theorists have emphasized, in somewhat different ways, the importance of speech and communication as forming the basis of action. These theories offer important insights into the pathologies that have generated the catastrophic breakdown that characterizes the current crisis in the Middle East. They draw attention to the need for a process of reconciliation—that is, the re-establishment of communication—committed to restoring a discourse directed towards restoring mutual understanding and opening the way to non-violent responses where conflicts arise. Stated in this way, the call for reconciliation is not a mere plea for the parties in conflict to talk to each other, or even to enter into formal negotiations. What is at stake is much more fundamental than this: it is a call for the construction of a process to establish some kind of translation function across apparently incommensurable discursive frameworks, effected in the domain of practical action. The possibility of such a process, and the structures underlying it, arise from the conditions of possibility of all human intercourse—that is, from the nature of ethics itself.

The radicalism of the claim that reconciliation is always possible, no matter how deep the rift between values or meaning-construction, should not be understated. In the western philosophical tradition theories of moral theories have commonly been understood as constructions that depart from ontological assumptions about the primal role of individual subjectivity (Husserl 2013). However, the current ethical crisis is not a purely theoretical event that reflects the free choices of disparate individual agents. Rather, the agents themselves are formed and directed by the ethical debacle, which constitutes the malaise with which they are afflicted.

The work of Emmanuel Levinas provides a compelling framework for understanding the foundational status of ethics, within which the analyses of Habermas and Arendt of the vicissitudes of communication within specific social and historical contexts provide specificity.

Levinas contends, against the western ontological tradition, that the appropriate starting point for the theorization of ethical conduct is the ethical bond itself (Levinas 1979). According to this perspective, the condition of possibility for all human relationships, including the formations of language and truth and subjectivity itself, is a primordial disposition of responsibility (Levinas 1979). In Levinas’ view ethics “as first philosophy” is the point of departure—logically, and even chronologically—for all the categorical constructions of philosophy and culture, including theories of ethics themselves. In the irreconcilable difference of alterity, he founds the fundamental relationship with the Other, which is the very beginning of, and the ultimate condition for, communication. Within the context of the ethical relationship, interactions are negotiated through linguistic exchanges that themselves preserve the mutual respect for and responsibility to others on which they are founded. The objective of these exchanges is to preserve, not to overcome, otherness; indeed, it is to resist attempts to do so:

The despair of impossible communication … marks the limit of all pity, all generosity, all love … But if communication thus bears the sign of failure or inauthenticity, it is because it is sought in fusion. One sets out from the idea that duality should be transformed into unity, that the social relation should end in communion … The failure of communication is the failure of knowledge. One does not see that the success of knowledge would in fact destroy the nearness, the proximity of the Other. (Levinas 1981, 103–104)

For Levinas, ethical language signifies, through the opposition between ‘‘the Said’’—the manifest content of language, which allows the imparting of information, knowledge, and meaning from one to another by means of representation—and “the Saying”—the dynamic process of exchanging signs, of addressing and being addressed, of active articulation—the “modality of the approach to the other person” (Levinas 1991, 142). Saying, therefore, is “not a game.” It is “the proximity of one to the other, the commitment of an approach, the one for the other, the very signifyingness of signification” (Levinas 1991, 142). Language is therefore essentially an expression of a relationship before it is a vehicle for the transmission of ideas. Language as Saying discloses itself as a manifestation of responsibility prior to taking the form of “the truth-that-unites” (Levinas 1991, 142).

It is important to recognize that from this viewpoint, while ethical discourse is based on communicative exchanges between individuals, such exchanges depend not just on shared assumptions, which can be linguistic, conceptual, or ethical, but that the assumptions themselves are outcomes of a more fundamental connection, here characterized as the possibility of ethics itself. As we shall argue, this is fundamental for the Israel-Palestine conflict and may be seen as one of the sources of optimism and hope.

At the risk of repetition, to re-state these points in another way, it is not the apparent agreed conclusions that makes ethical discourse possible but the dynamic interchange itself, which rests not on similarity but on communication across a wide range of differences defined by culture, gender, race, nationality, and other characteristics. Indeed, it may be observed that if ethical discourse required, or was founded on, sameness, it would have nothing to offer, and could produce nothing. It is only the fact of difference and the novelty to which it gives rise, that provides ethical intercourse with its radical capability of traversing the discontinuities of discourse and meaning and tracing paths of intelligibility between systems of meaning production and representation. Indeed, difference premised on the ontological, originary force of ethics is the motor that drives us into new territories of meaning and sense and enables the overcoming of antagonisms that formerly appeared irreconcilable (Boundas 1985).

From this point of view, the complexity of the ethical endeavour associated with struggles to repair the threads of dialogue that have been broken becomes apparent. The “solution” to disagreements with ethical content does not lie in purely theoretical constructions erected on the basis of rational, religious, or other precepts to justify one party’s position rather than another. Ethical agreement is not established through the application of brute force—even the brute force of reason—but through dynamic and generous communicative exchanges that mobilize difference to create new, hitherto unimagined, pathways for understanding. In other words, ethical conflicts cannot be resolved by the exercise of power or authority. Rather, by precipitating ongoing processes of fecund dialogue arising out of the fundamental conditions of ethics itself they can give rise to novel possibilities of meaning and hitherto unimagined regions of shared understanding. In the context of actual conflicts, such as the one in the Middle East, therefore, the ethical problem is not a pure abstract need to construct elaborate arguments to justify the preference for one course of action over another. Indeed, by turning what purports to be communication into polemical deployments of power, the accumulation of such constructions, developed by one side or another, can actually obstruct communication and provide a barrier to resolution. Multiple philosophical texts fall into this category—for examples, those that seek to provide definitive expositions about the “ethics of war,” “moral equivalence,” “relativism,” the “right to self-defence,” “ethics of torture,” and many other familiar academic topics (Frowe 2022; Evans 2007; Rodin 2004; Shorten 2011).

To avoid allowing ethical discourse to be suborned into yet another weapon in the conflict, reconciliatory dialogues seek to foster authentic processes of mutual sense-making, of sharing meaning construction, of translation between opposing world views, on the basis of which the protagonists can begin to imagine new narratives for understanding and cooperation. As Habermas puts it, in this process moralities must “emphasise the inviolability of the individual by postulating equal respect for the dignity of each individual,” but they must also protect “the web of intersubjective relations of mutual recognition by which these individuals survive as members of a community” (Habermas 1990, 200). In this sense, “all moralities coincide in one respect: the same medium, linguistically mediated interaction, is both the reason for the vulnerability of socialised individuals and the key resource they possess to compensate for their vulnerability” (Levinas 1991, 102).

## Reasons for Hope

The understanding of the nature of the conflict in Israel and Palestine as a breakdown in communication within a framework encompassing pre-existing ethical relationships, historically conditioned and subject to multiple external forces, potentially opens the way not only for a degree of optimism in relation to the future but even to practical strategies towards peace.

Possibly to the surprise of many external observers, but undoubtedly familiar to all parties in the region, a vast array of rich resources exists that could potentially provide a basis for the healing of the fractured ethical bonds. Pieced together meticulously over decades, these resources offer the possibility of disproving common assumptions about the conflict and building the basic relationships needed for a reconciliation process to unfold. Indeed, paradoxically, some of the forces unleashed on October 7 may emerge as productive, rather than as unequivocally noxious. For example, arguably, the events and their aftermath may have driven home to communities on both sides the failure and destructive force of pre-existing policies and strategies. They may be seen to demonstrate the weaknesses of both the political Islamism of Hamas and the divisive policies of the Israeli right-wing. Potentially, for these reasons (or, at least, hopefully), they may generate a recognition that confrontation and violence have failed and that there is an unavoidable need to seek an alternative way forward (Maoz and Ron 2016; Bar-Tal 2000, 2013; Bekerman, Maoz, and Sheffel 2006).

What exactly are these resources? They comprise an inventory of values, aspirations, hopes and dreams, built up over many years of shared experience and struggle, of Palestinians and Israeli Jews from all walks of life. They reflect a complex history of cooperative endeavours, creative partnerships, and alliances built around a joint search for security, justice, truth, and ethics (Cohen 2019).

The creators of the inventory come from all parts of Israeli and Palestinian society. On the Israeli side they include people from the border kibbutzim who were caught up in, and in some cases tragically become victims of, the events of October 7. This sad fact does not invalidate or negate their aspirations for peace and reconciliation but rather affirms them. Countless other Israelis have worked hard for reconciliation, often standing in opposition to governments and religious or community groups supporting division and violence (see also Golan and Orr 2012). At the same time—despite the impression cultivated by the prominence of the militant discourses of Hamas—Palestine is a sophisticated secular society with a refined culture that highly values peace and a highly educated population, even if the nature of educational practice is not immune from criticism (Sukarieh 2019).

On both sides, a great many individuals and organizations have been active in struggles for peace, justice, human rights, and the fair distribution of wealth. These organizations are readily identifiable and include charities, medical and healthcare associations, civil society cooperative groups, economic partnerships, parent organizations, religious groups, political grass roots movements, environmental groups, and cultural associations, among others. Although the actual nature of the groups varies between Israel and Palestine, reflecting the different cultural and political circumstances, both have since their inception been sites of vigorous ferment and active engagements.

Literally hundreds of civil society groups on both sides can be named. A brief and partial list of NGOs devoted to the cause of peace in Palestine^4^ and Israel^5^ is provided in the notes. Here, for the purposes of illustration I mention only a few.^6^ On the Israeli side there are peace and anti-war groups (IPHR, Women Wage Peace [see also Svirsky 2004]), human rights groups (B’Tselem, Standing Together), groups of families affected by the conflict (Parents Circle), religious partnerships (Rabbis for Peace), diaspora activist groups (IPCRI, NIF, Centre for Protection of Democracy), educational groups (Kids4Peace, Kids Creating Peace, Rossing Centre), charities (Humans without borders, Project Rozana), economic partnerships (Centre for sustainability) and many others. Israeli peace activists and civil society groups for years have worked on plans for reconciliation—even to the point of producing maps of Jerusalem in which proposed or imagined parallel governance arrangements are set out. Many of these groups either include Palestinian people directly or work closely with them to coordinate their activities.

On the Palestinian side, the rise of political Islam in Gaza has coloured the nature of civil society activism and in some ways, perhaps ironically, enhanced it. Nonetheless, many organizations similar to the Israeli ones have continued to flourish and, even in the face of risk, cooperation has been maintained. There are culturally based civil society partnerships, stimulated in part by the failure of local governance processes in Gaza. Many similar organizations exist: women and children’s support (Culture and Free Thought Association, Adam Association, Defence of Children International, Women for life), medical (Gaza Community Mental Health Program, Palestine Medical Relief Association, Alasol Altayaba Society), economic think tanks (PalThink, Seeds Association), youth support groups (Aida Youth Centre, Palestine Youth Action Centre, Youth and Environment Association, Youth without Borders), community support for specific populations (Al Mostaqbal Association, Development of Orphans), educational foundations (Al Nayzak), environmental groups (Palestinian Friends of the Environment, Youth and Environment Association) and many charitable organizations devoted to undermining the palpable antipathy that Israelis experience (Arab Network for Tolerance, International Peace and Cooperation Centre, Project Hope, Ta’awon for Peace and development, Palestinian Initiative for peace and democracy). The cultural renaissance has drawn attention to the damaging effects of occupation and colonization and the need for a cultural renewal. Ironically, one of the legacies of the incapacity of Hamas to govern Gaza effectively was a florescence of civil society groups based on mutual care and generosity. The sense of siege, while undoubtedly fuelling hostility to Israelis, also created a sense of solidarity and a vision of a society based not on war and persecution but on an ethos of mutual responsibility and care.

Added to these lists of indigenous NGOs, international organizations, involving many individual Palestinians and Israelis, have also been active. These include the Red Cross/Red Crescent/International Committee of the Red Cross, Save the Children, Médecins Sans Frontières, Action Aid, Care, World Vision, Oxfam, and many others. While these organizations are by no means free of internal biases they also are important contributors to discourses around reconciliation.

In addition to the impressively rich civil society institutional landscape there are innumerable inspiring individual stories of cooperative relationships and projects, initiated from both sides—including stories of resistance against the oppressive regimes by their own citizens. In many of these cases individuals have risked their lives to protest the actions of the Israeli army and police or the Hamas authorities. Sites of resistance have developed in all areas of activity, including the domains of culture, economics, environment, religion, education, healthcare, and sport. There has also been a vast outpouring of challenging cultural works in the fields of literature, theatre, cinema, and music. Gaza has a thriving rap culture, which provides a forum for young people to engage in their own kinds of dialogue, both to clarify their own views and to express them publicly. There are high levels of awareness on both sides of the implications of the environmental challenges associated with climate change, and multiple grass-roots partnerships have formed around sustainable horticultural and water conservation techniques. Sporting teams have sought to traverse the boundaries set by the political regimes. Economic partnerships have proved fertile and productive. Educational programmes have emphasized the need for respectful discourse through which differences can be negotiated in the fields of culture and values.

The massive ferment in both Palestine and Israel referred to above, which has involved countless individuals, has arisen and flourished in spite of, or perhaps because of, the oppressive regimes that have acquired power on both sides. It should not be forgotten that these have been constructed on the basis of links between communities in the region that stretch back to antiquity. Indeed, it is important to remind ourselves that the physical separation of the people of Palestine and Israel is only a recent phenomenon, created and enforced in the present century—often violently—by the regimes on both sides.

These partnerships signify a number of things: a rich history of cooperative struggles for peace; a shared commitment among many people on both sides; multiple experiments and experiences that can be mobilized as a basis for future initiatives and as learning experiences to guide action. Together, they comprise a profound resource that can be drawn upon should the possibility of a future attempt at reconciliation arise. However, it is important to emphasize that they do not in themselves point uniquely to a pathway ahead. For this, further analysis is needed that provides understanding and clarity about the cultural, political, and other sources of the current crisis and an indication about the kinds of changes that are likely to be needed to overcome the overwhelming obstacles that have arisen.

## Reconciliation Requires Identification of Both Common Interests and Obstacles to Communication

Struggles for reconciliation are based on fundamental assumptions about ethics as the basis on which all human intercourse is founded and the interruption of the communicative processes according to which such intercourse is transacted. They stand in opposition to discourses that harden existing positions and exacerbate communication blockages.^7^ They avoid basing future actions on judgments about past events but instead seek to learn from past failures in the hope of generating novel possibilities and opportunities for ethical consensus.

The restoration of the elements of discourse that incorporates creative and respectful communication and dialogue across difference must depart from a common starting point. Shared interests are not difficult to identify: they are implicit in the ethical assumptions that are built into the civil society movements referred to above and reflect actual engagements between individuals in both communities immersed in common lifeworld concerns saturated with values and grounded in actual relationships. The common starting point reveals two key conditions of possibility: on the one hand, they imply a degree of mutual commitment and respect or responsibility that is the foundation for all ethical thought and action; and on the other, based as they are on active engagements within and between communities, they embrace an unsuppressible acknowledgment of difference, to be negotiated though dialogue. Both assumptions, which are basic premises of an ethic based on reconciliation, stand in contrast to the reified categorical constructions of philosophical ethics (Husserl 1970). Together, they make available a common language based on shared values and aspirations and hopes and dreams (Bekerman, Maoz, and Sheffel 2006).

But the common interests cannot conceal the deep structural impediments to change. Indeed, the proliferation of peace organizations may itself suggest that the need exceeds the volume of successful outcomes. To disentangle the forces unleashed in the current catastrophe and lay out potential strategies for tangible steps forward, an awareness of the origins of the conflict is essential—including its historical and political context as well as its deep cultural roots (Serres 1968).

It is obvious that the sources of the current conflict extend far beyond October 7, 2023. The pain and frustration of Palestinian people over years of occupation and denial of rights was self-evidently a driving force. The desperation created by repeated failures to progress towards a future of peace has undoubtedly taken its toll on both sides. The refusal of dialogue, the expressions of contempt, the burden of mounting injustice on both sides, created a dangerous mixture in which an explosion was predictable.^8^ On the Israeli side, attempts at reconciliation had been obstructed by demographic changes, an inexorable move of successive governments to the political right, increasing internal social and religious divisions, and the growth of a culture of corruption that fed on perpetuation of the conflict. On the Palestinian side, a similar sense of betrayal arising from another corrupt and self-serving political leadership engendered a sense of hopelessness, and humiliation, and a desperate sense that there was nothing to lose by violence. After so many years of unrelenting threat, danger, uncertainty, and eruptions of actual violence many people on both sides had retreated from hope into fatalistic indifference or even callous cynicism. The sense of moral aridness linked to the ever-present sense of imminent disaster created a discursive wasteland in which attempts to advocate for values or value-based practices for many no longer carried conviction.

If progress towards peace and reconciliation is to occur all these obstacles will have to be confronted.

## The Sensitive Question of the Origins of Totalitarianism and the Lessons Thereof

As impressive as the institutional and political failures may be, on their own they are insufficient to explain the complete collapse of moral discourse that characterizes the current conflict. The sheer horror of the events—the indiscriminate killing of civilians, the targeting of health infrastructure, the cruel disregard of restraints supposedly mandated by international norms—arguably goes well beyond the bare contingencies of corruption and internecine discord. The possibility of an ethical singularity of such magnitude raises the question of even more fundamental factors operating deep within the culture. If we are to have any chance of progressing we need to understand how such things can be possible at all.

Here the work of Levinas, Habermas, and Arendt can once again offer assistance. Indeed, Levinas and Arendt in particular were both pre-occupied with another major historical event in which ethical boundaries were similarly dissolved and unrestrained atrocities were unleashed. Here, an immediate disclaimer is needed. By referring to the horrors of Nazism there is no intention crudely to identify any party in the current conflict with the hateful ideology of National Socialism or to engage in accusations with polemical intent. Rather, the purpose is to return to the deep analyses undertaken by—mainly Jewish—thinkers about a similar case of moral rupture in order to learn the lessons of the past, this time in the hope of avoiding yet another such catastrophe in the future.

Zygmunt Bauman—another Jewish theorist of the Holocaust (Bauman 2023)—similarly emphasized the need to reflect systematically on its sources without imposing assumptions or limitations. He criticized attempts by the Jewish establishment to resist attempts to expropriate the injustice that the Jews and the Jews alone suffered, or to separate the suffering, this injustice or lessons it may make available to us, from the Jewish state. “This unholy alliance,” Bauman wrote:

… effectively prevents the experience it narrates as “uniquely Jewish” from turning into a universal problem of the modern human condition and thus into public property. Alternatively, Auschwitz is cast as an event explicable only in terms of the extraordinary convolutions of German history, of inner conflicts of German culture, blunders of German philosophy or the bafflingly authoritarian national character of the Germans with much the same parochializing, marginalizing effect … One would expect this strategy to be a favourite form of self-defence: after all, it obliquely reaffirms and reinforces the etiological myth of modern civilization as a triumph of reason over passion, and an auxiliary belief in this triumph as an unambiguously progressive step in the historical development of morality. (Bauman 1991, 140–141)

In his vivid analysis of what he referred to as the “philosophy of Hitlerism,” Levinas concluded similarly that the phenomenon of Nazism was not a gratuitous crime committed by deranged individuals, or even an expression of a unique historical and social set of circumstances of a particular society at a given time. Rather, he considered it to expose a latent vulnerability in western culture. For him, this vulnerability lay in pathologies of being, typified by a shift from the primacy of ethics to discourses based on individualism and freedom. In an updated preface to this work, written in 1994, he wrote that his core argument

… stems from the conviction that the source of the bloody barbarism of National Socialism lies not in some contingent anomaly within human reasoning, nor in some accidental ideological misunderstanding. This article expresses the conviction that this source stems from the essential possibility of elemental Evil into which we can be led by logic and against which Western philosophy had not sufficiently insured itself. (Levinas 1990, 63)

For Levinas, Hitlerism disclosed a possibility that the Western tradition carried within it. This possibility was inscribed within the ontology of a being concerned with being. It is the fate of our times that such a possibility continues to threaten, years after the demise of National Socialism itself. We must ask ourselves similar questions, he argues, about the ethical assumptions commonly deployed in the modern world. “Does the subject arrive at the human condition prior to assuming responsibility for the other man in the act of election that raises him to this height?” (Levinas 1990, 63)

Arendt in turn, in her studies of the origins of totalitarianism and the dynamics of the human condition, also found identifiable forces that persisted in contemporary society that were fundamental to the rise of Nazism. In her analysis she drew attention to pathologies in the structure and use of language that arise as preconditions for a similar moral crisis. Specifically, she described the instrumentalization of speech and thought that turned them into tools of conformity and obedience. The suppression of dissenting voices limits diversity of thought and expression, undermining the very essence of free and open communication. Propaganda and manipulation of language through which language becomes a tool for mass persuasion and manipulation—that is, as a tool not for the development of a practical consensus but as a weapon. For her, these changes led inexorably to a corollary: the loss of truth, or at least the loss of a standard by which truth or falsity could be determined. Repetition of propaganda for the purposes of achieving an instrumentalist objective undermine the ability to discern the difference between truth and falsity. Together, these strategies created the isolation of individual citizens, the dissolution of the public sphere and the inability of a dialogical process to be sustained. The path from the atrophy of speech to naked violence is a direct one (Arendt 2017):

Without the disclosure of the agent of the act, action loses its specific character and becomes one form of achievement among others. It is then indeed no less a means to an end than making is a means to produce an object. This happens whenever human togetherness is lost, that is, when people are only for or against other people, as for instance in modern warfare, where men go into action and use means of violence in order to achieve certain objectives for their own side and against the enemy. In these instances … speech becomes indeed “mere talk,” simply one more means toward the end, whether it serves to deceive the enemy or to dazzle everybody with propaganda; here words reveal nothing, disclosure comes only from the deed itself … . (Arendt 2013, 180)

The importance of a critical understanding of the significance of this process cannot be overstated. “We are perhaps the first generation,” Arendt declared, “which has become fully aware of the murderous consequences inherent in a line of thought that force one to admit that all means, provided they are efficient, are permissible and justified to pursue something defined as an end” (Arendt 2013, 229; see also Dietz 2000).

In Levinas’ words, under such circumstances “thought becomes a game” when an abstract notion of freedom undermines and overwhelms truth and conviction. “Civilization is invaded by everything that is not authentic” (Levinas 1990).

Habermas’ analysis, consistent with these trenchant diagnoses, adds further procedural insights and draws attention to the damage sustained by the public sphere, which in normal times provides a necessary protective mechanism for ethical conduct. For him, as for the other two, the instrumentalization of communication is crucial, because it shuts out dissenting voices and silences criticism. The loss of critical public discourse opens the way for the propagation of authoritarian and racist ideologies, which are often supported by allegations about the undermining of national unity and culture. These pathologies in turn produce a structural crisis, referred to as a “legitimation crisis,” in which the moral authority of the ruling institutions, along with the broader framework according to which they are supposed to be regulated, are further eroded (Habermas 1973).

Levinas, Arendt, Bauman, and Habermas are not the only thinkers to have argued that the moral crisis of Nazism arose systematically out of a system of cultural and economic imperatives comprising deep philosophical precepts and invoking characteristic pathologies in the media of communication and cultural exchange. The question they collectively pose, however, is a common one: if the ethical wasteland of National Socialism was not an exceptional event but expressed innate tendencies within western culture, how are such tendencies to be identified and controlled in other settings? Further, it is then no longer surprising that similar forces persist in contemporary society—and indeed, have recognizably manifested themselves in both Israeli and Palestinian societies. In other words, both societies display the same susceptibilities within their cultures that constituted the conditions of possibility of Nazism—and those susceptibilities have even come to fruition in the worst possible way.

This conclusion may come as a shock to many but, on both conceptual and empirical grounds, cannot be avoided: it is possible that the ethical maelstrom unleashed on October 7 merely recapitulated latent tendencies that had been lurking deep within the hearts of both Israeli and Palestinian cultures.

## Is a Reconciliation Process Possible?

It is in this difficult and fraught setting that the question of the possibility of constructing a reconciliation process must be considered. I have argued that while the two peoples share many values and a common desire for peace, historical and political factors pose significant barriers, added to which is an inherent vulnerability located within the deep structures of both cultures.

It is the claim of this essay that, in spite of these formidable obstacles, hope for a reconciliation process remains alive, drawing for its possibility on the rich, albeit fragile, civil society infrastructure that has been assembled so painstakingly over the years. The fact of such an infrastructure is, I believe, evidence of fertile ground for the establishment of a living reconciliation process. However, this is not to suggest that such a process is likely to arise spontaneously or that it could be sustained independently of vigorous assistance and support from both within and outside the communities concerned.

While the analyses of Levinas, Habermas, Arendt, and others provide guidance about how to proceed they do not guarantee that the parties to the horror will actually agree to participate or that, if they do, the process will be successful. Levinas’ diagnosis of the substitution of the dialogical relationship by philosophical frameworks that favour what he called the crude “immanence” of religion, race, or nationality, provides one potential strategy, to which he refers as a return to the “essence of Judaism,” defined as a “restless going beyond that is open to difference and opposes a refusal of open dialogue” (Levinas 1990, 64–65), an “immediate sentiment of the contingency and insecurity of the world, a restlessness of not being at home and the force to leave it” (Levinas and Portal 1938, 6–7; Levinas 2020, 739–740). Habermas and Arendt offer other suggestions which provide varying degrees of practical guidance. However, ultimately, these and other attempts at reconciliation will depend on the good will and determination of all relevant parties.

## Technics of Reconciliation

If it is agreed that a reconciliation process should be attempted how should it proceed? What would be its objectives? How should it be facilitated or supported? What would be the conditions for its success? While it is neither possible nor appropriate for these fundamental questions to be addressed in detail at this time, some limited comments can be offered about the broad framework of a reconciliation process and conditions for its success (Rothfield, Fleming, and Komesaroff 2008; Komesaroff, Kath, and James 2011).

First, regarding the structure and dynamics of reconciliation discourses, it is important to recognize that these do not follow fixed formulae or obey irrefragable rules. Rather, the commitment to dialogue that drives the process forward can manifest itself in multiple ways, employing as necessary both formal and informal strategies in the search for common ground. Nonetheless, there are some constant features. Reconciliation praxis takes the form of linguistically mediated symbolic action that draws for its raw material on the shared lifeworld experiences of the participants. The latter encompass the dense plenum of experiences acquired in the course of everyday life in civil society contexts. The heterogeneity of perspectives, responses, attitudes, and beliefs is accepted as a premise that reflects the underlying ontological condition of difference and the dialogues themselves seek to deconstruct instrumentalist formulations in order to replace them with open, fluid explorations directed towards shared areas of meaning. This coming into existence of new forms of articulation—if it occurs—allows what had previously been unsayable to be rendered into the public space as, a new active saying.

Second, in this way, reconciliation dialogues may be seen as seeking to mark out a translation function between disparate meaning systems. To achieve this they need to overcome the distortions of language that limit its communicative effect, especially by converting it into a weapon or tool for the achievement of a preconceived end rather than cultivating the active deployment of speech for non-utilitarian outcomes. The restoration of communicative possibilities requires an acknowledgment of the importance of procedural—that is, pragmatic—norms that comprise conditions of possibility of dialogues that could ultimately achieve consensus. However, it is also recognized that cross-discursive communication has to draw on multiple modalities of cognition, affect and linguistic expression, thereby precluding formal processes of translations and hermeneutic rules or algorithms to guide its realization (Laclau 2014).

In this way, by identifying and addressing blockages of communication and tracing out a pathway of understanding between divergent systems of thought, reconciliation processes seek to create conditions in which the healing of the ethical rupture associated with a conflict—that is, the loss of trust and respect, the dissolution of a mutual sense of responsibility and care—can begin to be healed. Of course, these are preconditions only, because the material circumstances of inequality, injustice, and violence must also be overcome. However, the two are interdependent, because no formal political settlement can be secure without the achievement of a process of mutual understanding, while the re-establishment of dialogue can only take effect if it reflects actual changes in lifeworld experiences.

Third, further to the last remark, in particular settings, such as the Israel–Palestine one, reconciliation discourses must address actual, factual circumstances and draw on real historical experiences. The latter include common goals and shared purposes, as well as differences in culture, philosophical disposition, and lifeworld strategies. They can therefore be both unifying and divisive, as they seek to remove the obstacles that have been erected in systems of communication over many years, by identifying and challenging distortions in the use of language, barriers to open dialogue, and the mechanisms for maintaining separation between individuals and communities. It is here that the historical inventory of cooperative relationships directed towards the achievement of shared goals and values in many areas will offer a powerful resource.

Finally, at least four specific conditions would need to be satisfied for any reconciliation process in Israel–Palestine to achieve even limited success: (i) there must be a favourable social and political climate, including a substantial groundswell amongst both populations in support of a non-violent resolution to the long-standing conflict; (ii) a formal legal framework will be required to protect public and private conversations and provide for the assistance of external facilitators; (iii) adequate support must be available from outside the region, including from international, national, and foreign civil society organizations; and (iv) the capacity must be available to convert discursive advances into enduring lifeworld changes through sustainable partnerships with defined purposes and tangible outcomes. All these conditions, but especially the last one, can be facilitated by reactivating multifaceted engagements between the presently estranged communities—for example, through the encouragement of collaborations and partnerships in the domains previously mentioned, comprising art and culture, sport, education, healthcare, environmental protection, and business.

It is obvious from this discussion that if any reconciliation process in Israel-Palestine—the mere possibility of which is at present purely hypothetical—is attempted, its outcomes will depend on multiple variables and its outcomes will be unpredictable. As a dynamic commitment to open dialogue, an ethic of reconciliation contains no guarantee of success or even a clearly defined criterion by which success might be judged.

## A Cautious Concluding Comment

In these dark times, when for many all hope seems to be lost, it is important to remind ourselves of the resources for peace and reconciliation, painstakingly assembled over many decades, that, despite the obstacles, remain tantalizingly within grasp. After all the pain and suffering, if the choice be made, a way forward always remains a possibility. That path, however, will inevitably be fraught and difficult. It will require a radical questioning of assumptions that have come to be taken for granted on both sides. It will require transformations in the regimes of power and a shift of focus, from antipathy and hostility to common goals and shared purposes. It will entail the fostering of a public domain of trust and respect, of appreciation of difference, and an openness to new ideas and diverse cultural perspectives.

It was once said that it is only for the sake of those without hope that hope is given to us.^9^ It is possible that today this insight is more applicable than it has ever been.

## Footnotes

1 As mentioned, many of the claims about current attitudes in Israel and Gaza are based on personal observations and discussions; in these cases individual sources are not cited.

2 This statement is based on personal communications.

3 These are too many to mention but for the purposes of this essay the following thinkers are of particular relevance: Weber, Adorno, Wittgenstein, Habermas, Arendt, Chomsky, Nietzsche, Bauman, Levinas, Lingis, Austin and Searle.

4 Here is a partial and selective list of NGOs in Israel and Palestine directed towards the service of peace: *Palestine*: Adam Association for the development of family and society; Aida Youth Center; Ajyal Association for Creativity & Development (ACCD); Aknaf al-quds charity institute for women’s empowerment; Al Mostaqbal Association for Violence Victims’ Care; Al Nayzak Organization for Supportive Education and Scientific Innovation; Al_Qarara Society Center for social development / جمعية مركز القرارة للتنمية الاجتماعية; Alasol.Altayba Palestinian Society for Cancer Care - TPSFCC ; Al-Ferdous Association for Women and Child Development ; Alkarma Cultural Forum - Bethlehem; ALRazi Cultural & social Association - جمعية الرازي للثقافة والمجتمع; Amwaj Association for Social Development an Improvment; Arab Network for Tolerance; Arab World Democracy and electoral Monitor (Al Marsad); Association for the Advancement of Children and Women ; Awtar Center Association for Artistic and Cultural Creativity. ; Benevolent Palestinian Child’s Friends Association; Building A Better World Association; CCRR Center for Conflict Resolution and Reconciliation; Center for Defense of Liberties & Civil Rights “Hurryyat”; Culture and Free Thought Association; Dalal Institution for Culture and Arts; Damur for Community Development; Defence for Children International/ Palestine; Development of orphans ; Gaza Association For Culture And Arts; Gaza Community Mental Health Program; Good assets for development and development; HEWAR for Peace & Development; Hiwar Center for Youth and Women’s Empowerment; HOPE FOR KIDS IN PALESTINE; Human Rights and Democracy Media Center “Shams”; International Peace and Cooperation Center; IPCRI—Israel/Palestine Center for Research and Information; Itijah; Life and Hope Association; North Society for Community Development and Development ; Palestine Future Society for Development and Democracy; Palestinian Center For Communication and Development Strategies; Palestinian Friends of the Environment Society ; Palestinian Institute for Human Rights; Palestinian Medical Relief Society (PMRS); Palthink; Project Hope (Humanitarian Opportunities for Peace and Education); Rose of Jerusalem; Seeds Association for development and culture; Ta'awon For Conflict Resolution; The center for democracy and community development; The Palestinian Center for Peace and Democracy “PCPD”; The Palestinian Centre for Rapprochement between People; The Palestinian Initiative for Promotion of Global Dialogue and Democracy- "MIFTAH"; The Palestinian Youth Action Center for Community Development (LAYLAC); Volunteering for Peace; Women for Life; Yes Theatre for Communication Among Youth; Youth and Environment Association ; Youth Without Borders Association—YWBA.

5 *Israel:* A New Dawn in the Negev; Achoti; Activism Festival; Adva Center; Afikim BaNegev; African Refugee Development Center; African-Israeli Stage; Al- Mashghal (The Factory)—Arab Center for Arts & Culture; Alaswar Institute for Cultural and Social Development; Alkhaimah—The Association for Education & Development; All for Peace Radio; Arava Institute for Environmental Studies; Beit Almusica; Beyond Words; Bina; Blue and White Rights (part of: The Institute for Zionist Strategies); B’Tsalem; Center for Advancement in Peace Initiatives; Challenge An Organisation for Conflict Transformation; Citizens for the Environment; Combatants for Peace; Community Advocacy; Creativity for Peace; Culture & Peace; Darkenu; Economic Empowerment for Women; EcoPeace; EcoPeace / Friends of the Earth Middle East; Ecotourism—Heritage- Education (EHE): A partnership for intercultural citizenship in 3 conflict zones; Engineers Without Borders; Friendship Village; Hagar: Jewish-Arab Education for Equality; Hand in Hand—Center for Jewish-Arab Education; Humans Without Borders; IFLAC: The International Forum for the Literature and Culture of Peace; IPCRI—Israel/Palestine Center for Research and Information; Israeli Physicians for Human Rights; Jaffa Institute; Keshev—The Center for the Protection of Democracy in Israel; Kids Creating Peace; Kids4peace Jerusalem; Mabat—Awareness in a Multicultural Society; Mahpach-Tagir; Meet—The Middle East Entrepreneurs of Tomorrow; Minds of Peace; Molad—the Center for the Renewal of Israeli Democracy; Morashtenu “Our Heritage”: The Charter for Democracy; Mosaica Center for Inter-Religious Cooperation; Negev Coexistence Forum for Civil Equality; Negev Institute for Strategies of Peace and Development (NISPED); Oasis of Peace; One Voice Movement; Other Voice; PARENTS CIRCLE - FAMILIES FORUM; Partnership for Social Change; PeacePlayers International—Middle East; Rabbis for Human Rights; Rossing Center for Education and Dialogue; Save a Child’s Heart (SACH) in Memory of the Late Dr. Ami Cohen; School for Peace (SFP), Neve Shalom / Wahat al Salam; Seeds of Peace; Service and Peace in the Community; Sha’ar la-Adam; SHATIL—Israel’s Leading Empowerment and Training Centre for Social Change Organizations; Standing Together; The Arab Jewish Community Center; The Association of Environmental Justice in Israel (AEJI); The Citizens’ Accord Forum between Jews & Arabs in Israel; The Heschel Center for Sustainability; The Peres Center for Peace; The Public Committee Against Torture in Israel; The Rossing Center for Education and Dialogue; The Van Leer Jerusalem Institute (VLJI); Three Faiths Forum Middle East; Tishreen; Tiyul-Rihla; TRUST - Emun; Ultimate Peace; Windows Channels for Communication; Windows Channels for Communication; Women Wage Peace; YEDID—The Association for Community Empowerment; Zochrot.

6 Partial lists of civil society organizations in the region committed to actions supporting reconciliation can be found at The Anna Lindh Foundation website (https://www.annalindhfoundation.org/members/non-governmental-organisations-information-and-support-centre); Secret Tel Aviv (https://www.secrettelaviv.com/magazine/blog/useful-info/peace-ngos-israel-database); Global giving (www.globalgiving.org/atlas/country); www.arab.org and others.

7 For this section see Bar Tal (2000 and 2013), Bekerman, Maoz, and Sheffel (2006), Maoz and Ron (2016), and Falah (2021).

8 In a personal conversation in 2016 with the author President Abbas of Palestine himself opined that the greatest threat facing both Palestine and Israel was the desperation of Palestinian youth denied opportunities other than violence to assert their agency. When this message was conveyed to the Israeli Home Office the offer for the two countries to work together to avoid a catastrophe it evoked incredulity.

9 Attributed to Walter Benjamin by Herbert Marcuse (1964).
